# Uygur type 2 diabetes patient fecal microbiota transplantation disrupts blood glucose and bile acid levels by changing the ability of the intestinal flora to metabolize bile acids in C57BL/6 mice

**DOI:** 10.1186/s12902-022-01155-8

**Published:** 2022-09-23

**Authors:** Chanyue Wang, Ye Wang, Hao Yang, Zirun Tian, Manli Zhu, Xiaoting Sha, Ju Ran, Linlin Li

**Affiliations:** 1 Pharmacological Department, Pharmacy College, Xinjiang Medical University, Urumqi, Xinjiang China; 2 Key Laboratory of Active Components of Xinjiang Natural Medicine and Drug Release Technology, Urumqi, Xinjiang China; 3 State Key Laboratory of Pathogenesis, Prevention and Treatment of High Incidence Diseases in Central Asia of Xinjiang Medical University, Urumqi, China

**Keywords:** Type 2 diabetes mellitus, Microbiota transplantation, Insulin resistance, Bile acids

## Abstract

**Background:**

Our epidemiological study showed that the intestinal flora of Uygur T2DM patients differed from that of normal glucose-tolerant people. However, whether the Uygur T2DM fecal microbiota transplantation could reproduce the glucose metabolism disorder and the mechanism behind has not been reported. This study was designed to explore whether Uygur T2DM fecal microbiota transplantation could reproduce the glucose metabolism disorder and its mechanism.

**Methods:**

The normal diet and high fat diet group consisted of C57BL/6 mice orally administered 0.2 mL sterile normal saline. For the MT (microbiota transplantation) intervention groups, C57BL/6 mice received oral 0.2 mL faecal microorganisms from Uygur T2DM. All mice were treated daily for 8 weeks and Blood glucose levels of mice were detected. Mice faecal DNA samples were sequenced and quantified using 16S rDNA gene sequencing. Then we detected the ability of the intestinal flora to metabolize bile acids (BAs) through co-culture of fecal bacteria and BAs. BA levels in plasma were determined by UPLC-MS. Further BA receptors and glucagon-like peptide-1 (GLP-1) expression levels were determined with RT-q PCR and western blotting.

**Results:**

MT impaired insulin and oral glucose tolerance. Deoxycholic acid increased and tauro-β-muricholic acid and the non-12-OH BA:12-OH BA ratio decreased in plasma. MT improved the ability of intestinal flora to produce deoxycholic acid. Besides, the vitamin D receptor in the liver and ileum and GLP-1 in the ileum decreased significantly.

**Conclusions:**

Uygur T2DM fecal microbiota transplantation disrupts glucose metabolism by changing the ability of intestinal flora to metabolize BAs and the BAs/GLP-1 pathway.

**Supplementary Information:**

The online version contains supplementary material available at 10.1186/s12902-022-01155-8.

## Background

Type 2 diabetes mellitus (T2DM) is a metabolic disease caused by either inadequate production of insulin or an improper response to insulin. The International Diabetes Federation estimates that there were 465 million people with T2DM in 2019 and that this number will rise to 578 million by 2035 [1]. Recent studies have suggested that intestinal bacteria play an important role in the development of T2DM and that different intestinal bacteria present at different stages of diabetes (e.g., prediabetes, T2DM or no diabetes) [2–4]. Our epidemiological study showed that the intestinal flora of Uygurs and Kazak T2DM patients differed from that of normal glucose-tolerant people [5] and found that normal glucose-tolerant Kazak faecal microbiota transplantation could improve glycolipid disorders by changing the bacterial composition responsible for producing SCFAs and activating the GPR43/GLP-1 pathway [6]. A recent study reported significant compositional differences in intestinal bacteria between impaired glucose tolerance, combined glucose intolerance, T2DM and normal glucose tolerance. Both prediabetes and T2DM showed a continuing decrease in the abundance of some butyrate-producing bacteria and the expression of related genes, indicating that changes in the intestinal flora reveal the progression of T2DM [7]. Differences in the intestinal bacteria of T2DM patients and healthy people include both the numbers and types of bacteria [8]. Dysregulation of the intestinal flora has been reported to contribute to the occurrence and maintenance of insulin resistance [9]. Metagenomic analysis has found moderate dysregulation of the intestinal flora in T2DM patients, and transplantation of feces from obese mice or obese people to sterile mice has been shown to cause obesity [10, 11]. Other studies have determined that the abundance of *Bifidobacterium* and *Akkermansia muciniphila* is inversely associated with metabolic disorders, low-grade inflammation, insulin resistance and T2DM [12, 13].

Intestinal bacteria produce free bile acids (BAs) through hydrolysis and metabolize primary BAs into secondary BAs through dehydroxylation, thereby regulating their balance. According to a recent report, different intestinal strains metabolize BAs of different types and with different efficiencies [14]. In addition, a cross-sectional study showed that differences in dietary intake were closely related to changes in total BA levels [15]. Another study showed that antibiotics altered the intestinal flora and improved the insulin sensitivity of high-fat diet (HFD) mice, with transplantation of the intestinal microbiota of mice after an antibiotic intervention to sterile mice reproducing the above changes, which were closely related to BAs [16]. The HFD causes significant changes in the intestinal flora and BAs, with significant increases in taurocholic acid (TCA), taurochenodeoxycholic acid (TCDCA), glycocholic acid (GCA) and glycogodeoxycholic acid (GCDCA) [17]. Antibiotics regulate intestinal *Lactobacillus*, increase tauro-β-muricholic acid (T-β-MCA) and ameliorate obesity and insulin resistance in HFD mice [18]. An intervention involving *Parabacteroides distasonis* was found to control weight, reduce blood sugar and improve lithocholic acid (LCA) and ursodeoxycholic acid (UDCA) levels [19]. BAs are important signaling molecules that transmit information through their receptors farnesoid X receptor (FXR), G-protein coupled receptor 5 (TGR5) and vitamin D receptor (VDR). BA synthesis and metabolism have been associated with multiple metabolic disorders, such as obesity, diabetes and chronic liver disease [20]. Another study [21] reported that the plasma concentration of deoxycholic acid (DCA) was significantly higher in T2DM patients than in a control group, that the level of cholic acid (CA) was higher in subjects with obesity than in healthy people and that DCA and CA were negatively correlated with insulin sensitivity. Accordingly, the objective of this study was to explore whether the dominant flora of T2DM feces could reproduce the glucose metabolism disorder and changes in BA levels that occur during the progression of glucose metabolism disorder in mice.

## Materials and methods

### Ethics statement

The use of human subjects and the C57BL/6 mice was sanctioned by Ethics Committee of the First Affiliated Hospital of Xinjiang Medical University (Urumqi, China; approval No: 20140212–113) and performed according to the Declaration of Helsinki. all methods were carried out in accordance with relevant guidelines and regulations. This study was carried out in compliance with the ARRIVE guidelines. Prior to sample collection, the donor consented (written) to participate and satisfied 2006 World Health Organization criteria for diabetes diagnosis and for inclusion and exclusion in human studies.

### Preparation of donor fecal microbiota transplantation fluid

One human subject was involved in the study. The donor was a 42-year-old male of Uygur nationality with a fasting blood glucose (FBG) of 10.41 mmol/L, postprandial blood glucose of 13.7 mmol/L, hemoglobin A1c of 9.60%, and symptoms of dry mouth, polydipsia and increased urination for 4 months with weight loss. He had no history of medication for diabetes. The donor had lived in Xinjiang for more than 20 years and had no history of hypertension, coronary heart disease, cerebrovascular disease, peripheral arterial disease, other endocrine diseases, tumors, or infectious disease. There was no relevant family history, history of surgery or blood transfusion, or history of drug or food allergy. The patient had not experienced any epidemics, lived in epidemic areas, or had contact with water-borne epidemics. He had not been a miner, had not lived in regions with exposure to high-fluoride or low-iodine, had no previous exposure to chemicals, radioactive substances, or toxic substances, and no history of drug abuse. Fecal samples were collected from the Uygur T2DM patient in the morning into sterile bags using sterile gloves. The bags were sealed, and the feces were transferred to the laboratory for processing within 1 h. For culture, a 5 g fecal sample was diluted to ten times its volume in 0.9% sterile NaCl solution and a 200 μL suspension added to 14 mL FS liquid medium (Qingdao Hi-Tech Industrial Park Haibo Biotechnology Co., Ltd., Shangdong, China) in a sterile tube and mixed thoroughly with a vortex mixer. The tube was then put into an anaerobic bag together with anaerobic gas reagent and anaerobic indicator, and the bag was placed in an anaerobic incubator chamber at 37 °C for 48 h. Distilled water and glycerin at a 1:1 ratio were mixed and autoclaved at 121 °C for 15 min. The sterile 50% glycerol solution and microbiota transplantation (MT) suspension were mixed at a volume ratio of 3:7 and stored at − 80 °C. For transplantation, we defrosted MT preparations at room temperature (RT), centrifuged them at 4000 rpm for 20 min, and suspended precipitate in 0.9% sterile NaCl solution.

### Animal experiments

Pathogen-free mice (1.5–2 month old C57BL/6 males), from Beijing Vital River Laboratory Animal Technology Co., Ltd., Beijing, China, were adaptively fed for 7 days in a pathogen-free environment of the Animal Center of Xinjiang Medical University. Ten mice/group were randomly assigned as follows: 1) normal chow diet (NCD), 2) normal chow diet + MT (NCD + MT), 3) HFD (HFD), and 4) HFD + MT (HFD + MT). The animal quality certificate number was NO 650007000078, and the animal feeding certificate number was SYXK (new)2016–0002. Beijing Huakang Biotechnology Co., Ltd. (Beijing, China) provided the high-fat feed(57 Kcal %).

The MT was intragastrically administered to mice at 0.2 mL once a day for 8 weeks. The bacterial count of the MT was about 3 × 10^8^ cfu/mL on a Maishi turbidimeter (Beijing Tianan Union Technology Co., Ltd., Beijing, China).

Mice underwent fasting blood glucose, oral glucose and insulin tolerance measurements when fasted overnight for 8 h(from 23:00 p.m. on the first day to 7:00 a.m. on the second day). Blood glucose levels were recorded using a glucose oxidase assay. The insulin tolerance test was performed after an intraperitoneal injection of 0.5 U/kg insulin (0.5 U/kg body weight). Blood glucose concentrations were measured at 0 min and 40, 90, and 120 min after insulin injection.

### 16S rDNA V3–V4 region sequencing

Fecal bacterial DNA in stool samples, collected at week 8 and stored at − 80 °C, were processed using the QIAamp Fast DNA stool kit protocol (Qiagen Co., Ltd., Shanghai, China). DNA concentrations were recorded using a Nano-1000 microspectrophotometer (Bio-Rad Life Medical Products Co., Ltd.). The *16S rDNA* V3–V4 segment was amplified using 341F, ACTCCTACGGGAGGCAGCAG and 806R, GGACTACHVGGGTWTCTAAT primers, using the following conditions; 94 °C for 4 min, plus 94 °C for 30 s, 55 °C for 30 s, 72 °C for 10 s, and 72 °C for 300 s over 30 cycles. Amplicons were purified and sequenced using the Illumina HiSeq 2000 platform. The Agencourt AMPure XP magnetic bead method was used to purify amplicons in elution buffer. An Agilent 2100 Bioanalyzer was used to detect the section scope and concentration of products, while HiSeq instrumentation was used to sequence qualified products according to the inserted fragment size.

After original sequencing data were cleaned, FLASH (v1.2.11) was used for sequence splicing by overlapping paired-end reads from double-end sequencing, and to tag highly variable regions. We used the USEARCH sequence analysis tool (v7.0.1090) to generate operational taxonomic unit (OTU) cluster or tags at > 97% similarity. Next, we compared representative OTU sequences with a species database annotated by Ribosomal Database Project Classifier (RDP v2.2) using a 0.8 confidence threshold.

### UPLC-MS material

The following reagents were LC/MS-grade; methanol (Sigma-Aldrich, St. Louis, MO, USA), formic acid (Tianjin Fuchen Chemical Reagent Co., Ltd. Tianjin, China), and acetonitrile (Fisher Scientific, Loughborough, UK). CA, LCA, DCA, tauroursodeoxycholic acid (TUDCA), chenodeoxycholic acid (CDCA), TCDCA and taurocholic acid-Na (TCA-Na) came from Beijing Solarbio Technology Co., Ltd., Beijing, China. UDCA, GCA, taurolithocholic acid-Na (TLCA-Na), glycoursodeoxycholic acid (GUDCA) and nateglinide were obtained from Shanghai Yuanye Biotech Inc. (Shanghai, China). T-β-MCA and β-MCA were supplied by SHANGHAI ZZBIO Co., Ltd., Shanghai, China.

### Standard solution and calibration curve preparation

Individual BAs and internal standard (IS) stock solutions were made up in methanol at 1 mg/mL. Mixed stock solutions of individual BA and IS were formulated in a 50:50 (v/v) methanol: water mix at 100 ng/mL. Subsequent solutions were generated in methanol: water (50:50, v/v). We prepared quality control solutions using appropriate dilutions of standard stock solutions. Separate BA standard and IS working solutions were generated by diluting stock solutions. Standard calibration curves comprising 10–500 ng/mL range concentrations were serially prepared from BA stocks. A 50 ng/mL IS concentration was maintained at all calibration points.

### UPLC-MS assays

The Acquity UPLC system (Waters, UK) attached to the Acquity UPLC BEH C18 (1.7 μm, 2.1 × 100 mm; Waters) column was used for UPLC analyses. Column and autosampler temperature = 40 °C and 8 °C, respectively. Sample injection volume = 3 μL. Eluent A = 0.1% formic acid in water. Eluent B = 0.1% formic acid in methanol. Rate of flow = 0.2 mL/min. We established the following 10 min elution gradient: in the first 1 min, the eluent comprised 80% A and 20% B, in the next 5.5 min, it was linearly altered to 30% A and 70% B, then eluent B was raised to 90% for 1 min, and finally 100% for 90 s. To condition the column, initial conditions were run for 60 s.

A Waters Xevo TQ-S MS (Waters) was used for MS; the ESI source was operated in negative-ion mode while operating in multiple reaction monitoring mode. Other parameters: source temperature = 150 °C, capillary voltage = 3.5 kV, and desolvation temperature = 550 °C. Highly pure nitrogen = curtain gas at 15 psi, nebulizer gas = 60 psi, and auxiliary gas = 60 psi. Collision gas rate = 0.25 mL/min. Using quanoptimizer software (Waters), the instrument automatically tuned collision energies, cone voltages, and transitions for individual BAs. Operating software was MassLynx 4.2 (Waters).

### Preparing samples

#### Serum

We thawed 50 μL mice serum samples on ice and spiked samples with 5 μL IS stock solution. Then, 300 μL cold methanol was added to precipitate proteins, tubes vortexed three times for 30 s each, and stored for 20 min at − 20 °C. After centrifuging for 15 min at 12000 rpm at 4 °C, supernatants were removed and set aside. We also performed a second BA extraction in 300 μL cold methanol, after which both supernatants were combined and dried under nitrogen. Residues were resuspended in 100 μL of a 50:50 (v/v) methanol:water mix and centrifuged as before for 1 min. All supernatants were passed through 0.22-μm membranes.

### Co-culturing BAs with fecal microbiota from mice after MT

TSB culture medium was prepared at 1.8 mg/mL in distilled water. Conjugated BAs (TCA, TCDCA, T-β-MCA, and GCA) and unconjugated BAs (CA and CDCA) were supplemented to the medium at 500 ng/mL final concentration. The samples were autoclaved at 121 ℃ for 15 min, cooled and divided into enzyme-free centrifuge tubes with 10 mL per tube. Then, 200 μL fecal bacteria solution (normal saline:fecal sample, 20:1) was mixed in tubes and stored for 3 days at 37 °C. Then, 300 μL cold methanol and IS (final concentration, 80 ng/mL) were added to 200 μL cultured samples to precipitate protein, and samples vortexed three times at 30 s each and incubated at − 20 °C for 20 min. After centrifuging tubes, supernatants were passed through 0.22-μm membranes.

### qRT-PCR

Ileal and liver tissue were RNA extracted in TRIzol and 1 μg RNA (500 ng/μl RNA) was reversely transcribed into cDNAs with the PrimeScript RT and gDNA eraser. An initial 2 min priming step at 42 °C was followed by reserve transcription steps according to manufacturer’s methods. QPCR was undertaken with a QuantStudio 6 Flex RT PCR detection system (ABI, USA) plus TB Green Premix. PCR parameters: 95 °C for 5 s, 60 °C for 31 s, and 72 °C for 30 s over 45 cycles. The relative gene expression levels were normalized using β-actin gene and quantified by the 2^−ΔΔCt^ method. Primers are listed (Table 1).

**Table 1:** RT-q PCR primers for each target gene

Gene	Sequence (5′-3′)	Tm
FXR	F: CGGCTGTCAGGATTTGTGC R: GAAGCCCAGGTTGGAATAGTAAG	60 °C
TGR5	F: ACTCTGTTATCGCTCATCTCATTG R: AAGCACTCGTAGACACCTTTGG	60 °C
VDR	F: CACCTGGCTGATCTTGTCAGT R: CTGGTCATCAGAGGTGAGGTC	60 °C
GLP-1	F: CAAACCAAGATCACTGACAAGAAAT R: GGGTTACACAATGCTAGAGGGA	60 °C
β-actin	F: GCACCACACCTTCTACAATGAGC R: GGATAGCACAGCCTGGATAGCAAC	60 °C

### Western blotting

RIPA buffer (100 mg in 1 mL) was added to ileal and liver tissue samples milled in liquid nitrogen. After 20 min on ice, sample tubes were vortexed for 20 s, centrifuged, and protein concentrations were determined using the PierceTM bicinchoninic acid (BCA) Protein Assay kit (Thermo, USA) and 30 μg protein (4 μg/μl protein) used for western blot. Quantified proteins underwent 10% SDS-PAGE, were blotted to polyvinylidene fluoride membranes, incubated with 5% milk/TBST for 120 min at RT, and refrigerated for 12 h at 4 °C with VDR, GLP-1, and GAPDH primary antibodies. The next day, membranes were subjected to IRDye whole IgG secondary antibodies. Protein bands were processed using a Licor Odyssey Scanner. The loading control was GAPDH.

### Data analysis

Data were analyzed by using SPSS 19.0 statistical software (SPSS, Inc., Chicago, IL, USA). Parameter differences among groups were evaluated using one-way ANOVA for normally distributed variables and Kruskal-Wallis test for non-normally distributed variables. Results are presented as means ± SEM. A General Linear Model-Univariate was used to evaluate the interaction between MT and the HFD. A *p* value < 0.05 was considered significant.

## Results

### MT induces glucose metabolism disorders in mice

**Fig. 1:**
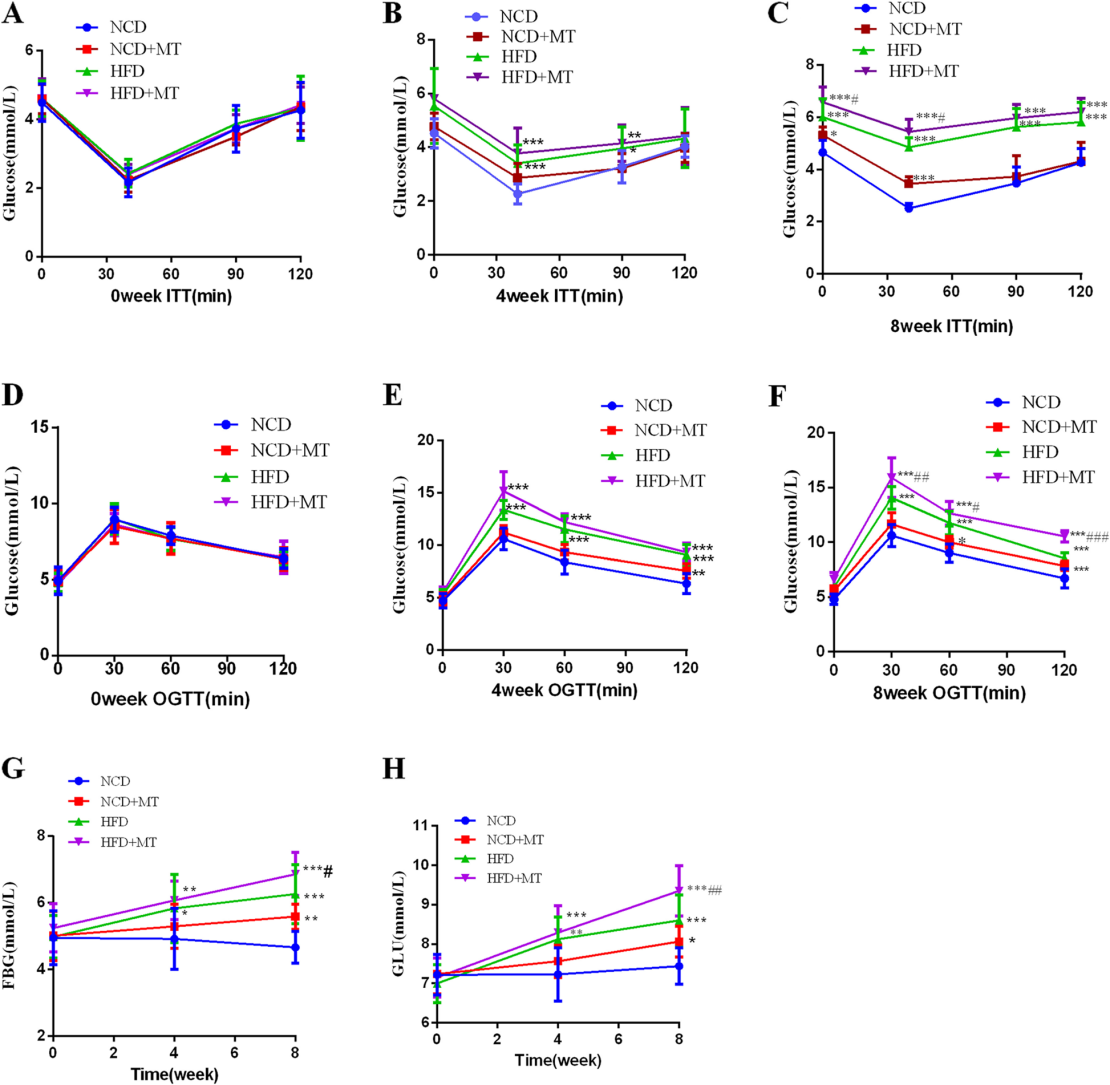
Effects of MT on glucose metabolism. Insulin tolerance test (**A-C**), oral glucose tolerance test (**D-F**), fasting blood glucose (**G**), random blood glucose (**H**). Results are expressed as the mean ± SEM (*n* = 8–10 per group). Compared with the NCD group, ^*^p < 0.05, ^**^p < 0.01, ^***^p < 0.001. Compared with the HFD group, ^**#**^*p* < 0.05, ^**##**^*p* < 0.01

As shown in Fig. 1, compared with NCD group, MT impaired insulin sensitivity and glucose tolerance (Fig. 1A–F). Besides, MT had significant effects on random blood glucose (*p* < 0.05) and fasting blood glucose (*p* < 0.01). Compared with the HFD group, random blood glucose and fasting blood glucose were significantly increased in the HFD + MT group at week 8 (Fig. 1G and H). The factorial analysis results also showed that there were synergistic effects between MT and the HFD on elevating 120-min glucose on the oral glucose tolerance test and reducing the glucose reduction rate of the insulin tolerance test at 40 min at week 8 (both *p* < 0.05).

### MT alter the composition of the intestinal flora

#### OTU cluster and abundance analysis

**Fig. 2:**
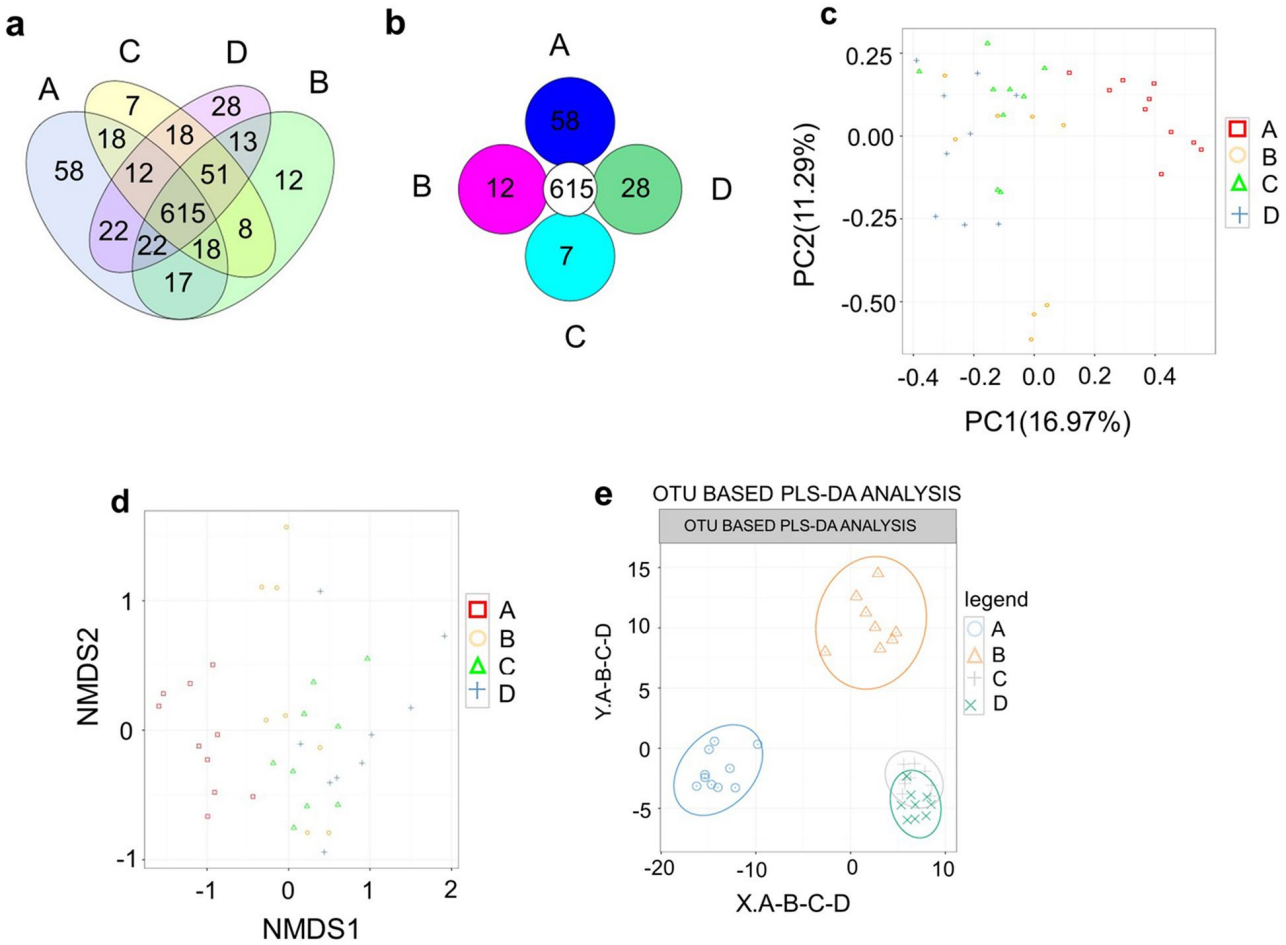
OTU cluster and abundance analysis. OTU Venn analysis (a), OTU Core-Pan analysis (b), Principal component analysis (c), Non-metric multidimensional scaling (d), partial least squares-discriminant analysis(e), (A) NCD(*n* = 10), (B) NCD + MT(*n* = 8), (C) HFD(*n* = 9) and (D) HFD + MT(*n* = 9)

Venn and Core-Pan diagram results showed that the NCD, NCD + MT, HFD and HFD + MT groups had 615 OTUs in common. The NCD, NCD + MT, HFD and HFD + MT groups had their own OTUs (58, 12, 7 and 28, respectively; Fig. 2A, B).

Principal component analysis (Fig. 2C), Non-metric multidimensional scaling (Fig. 2D) and partial least squares-discriminant analysis (Fig. 2E) found significant differences between the NCD and NCD + MT groups and the other groups. No significant differences were found between the HFD and HFD + MT groups. The results indicate that the HFD and fecal MT both had an impact on the composition of the intestinal flora.

#### Species composition and abundance analysis

**Fig. 3:**
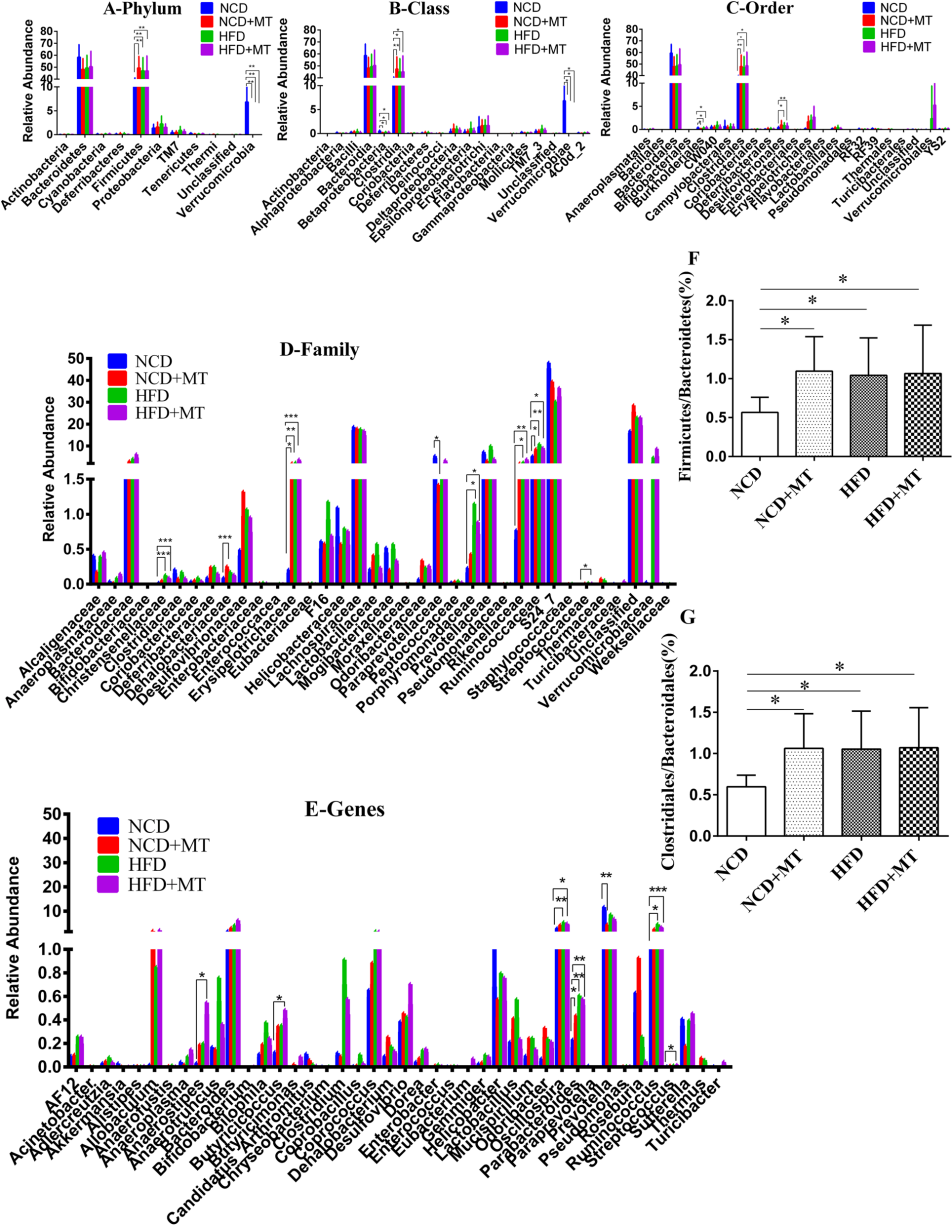
Influences of MT on the intestinal flora structure. NCD(*n* = 10), NCD + MT(*n* = 8), HFD(*n* = 9) and HFD + MT(*n* = 9). Compared with the NCD group, ^*^p < 0.05, ^**^p < 0.01, ^***^p < 0.001，

We examined the phylum, class, order, family and genus classifications of the bacteria in the microbiota. The phyla *Bacteroidetes*, *Firmicutes*, *Proteobacteria*, TM7, *Tenericutes* and *Verrucomicrobia* were abundant. Nearly 90% of the bacteria were members of the phyla *Bacteroidetes* and *Firmicutes.* The *Verrucomicrobia* population was significantly smaller and the *Firmicutes* population was significantly larger in HFD + MT mice than in NCD mice (Fig. 3A). In addition, both the HFD and MT significantly increased the *Firmicutes* to *Bacteroidetes* ratio (Fig. 3F).

At the class level, the intestinal flora mainly comprised *Bacteroidia*, *Clostridia*, *Verrucomicrobiae*, *Erysipelotrichi*, *Deltaproteobacteria*, TM7–3 and *Epsilonproteobacteria*. *Bacteroidia* and *Clostridia* occupied the largest proportion (almost 90%). Compared with the NCD, MT and the HFD significantly reduced *Verrucomicrobiae* (both *p* < 0.05) and *Betaproteobacteria* (both *p* < 0.05) and increased *Clostridia* (Fig. 3B).

At the order level, *Bacteroidales*, *Clostridiales*, *Erysipelotrichales*, *Verrucomicrobiales*, *Desulfovibrionales* and *Campylobacterales* were abundant. Nearly 90% of the bacteria were members of the *Bacteroidales* and *Clostridiales* orders. Compared with the NCD control group, both MT and the HFD significantly reduced *Burkholderiales* and increased *Clostridiales*. In addition, *Desulfovibrionales* was significantly larger in the HFD group (Fig. 3C). Moreover, both the HFD and MT significantly increased the *Clostridiales* to *Bacteroidales* ratio (Fig. 3G).

At the family level, both *Erysipelotrichaceae* and *Ruminococcaceae* were significantly larger in the NCD + MT group and in the HFD group. MT resulted in a significant increase in members of the *Dehalobacteriacea* family and a significant decrease in members of the *Paraprevotellaceae* family. The HFD increased the members of *Christensenellaceae, Porphyromonadaceae* and *Rikenellaceae* (*p* < 0.001, *p* < 0.05 and *p* < 0.05, respectively; Fig. 3D).

As for the genus level, compared with the NCD control group, both MT and the HFD upregulated *Parabacteroides* (*p* < 0.05 and *p* < 0.01, respectively). The *Prevotella* population was significantly lower in the NCD + MT group. The HFD significantly increased *Oscillospira* and *Ruminococcus*. The *Anaerostipes*, *Butyricicoccus*, *Oscillospira*, *Parabacteroides* and *Ruminococcus* populations were significantly larger in the HFD + MT group (Fig. 3E).

### Changes in bile acids metabolism in the recipient mice

**Fig. 4:**
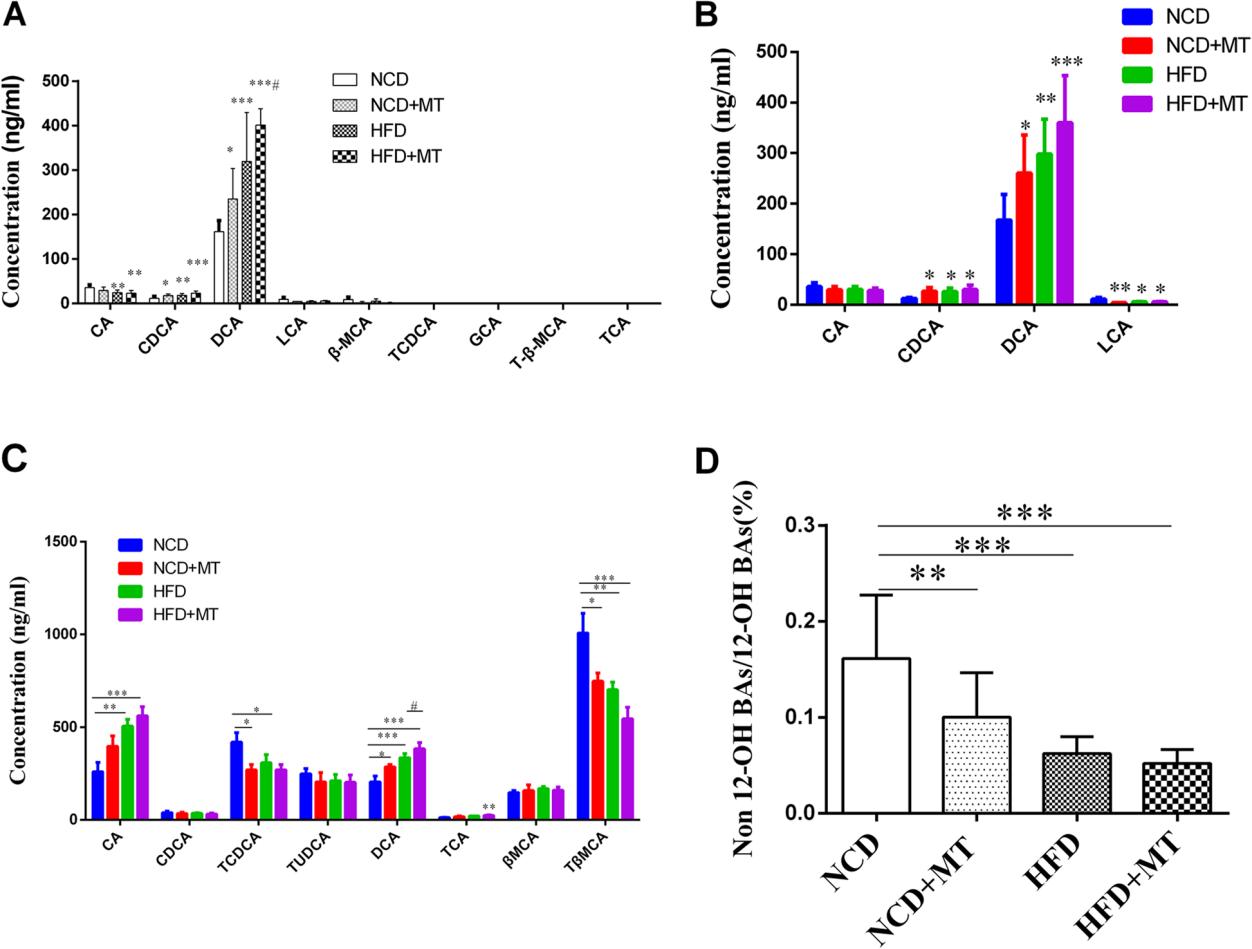
Changes in bile acids metabolism in the recipient mice. Ability of mouse intestinal flora to metabolize conjugated BAs after MT (**A**, *n* = 6 per group). Ability of mouse intestinal flora to metabolize unconjugated BAs after MT (**B**, *n* = 6 per group)**.** Effects of MT on plasma BAs (**C**, **D**, *n* = 8–10 per group). Results are expressed as the mean ± SEM. Compared with the NCD group, ^*^p < 0.05, ^**^p < 0.01, ^***^p < 0.001. Compared with the HFD group, ^#^*p* < 0.05

The ability of mouse intestinal flora to metabolize conjugated BAs results showed that CDCA and DCA were significantly higher in the MT group and HFD group and CA was significantly lower in the HFD group compared with the NCD group. In addition, compared with the HFD group, DCA was significantly higher in the HFD + MT group (Fig. 4A). The ability of mouse intestinal flora to metabolize unconjugated BAs results showed that CDCA and DCA were significantly higher in the MT and HFD groups and that LCA was significantly lower in the MT and HFD groups compared with the NCD group (Fig. 4B). MT increased the plasma DCA (*p* < 0.05) and reduced the plasma TCDCA and T-β-MCA levels (both *p* < 0.01). In addition, compared with the HFD group, DCA was significantly higher in the HFD + MT group (Fig. 4C). Plasma non-12-OH BA:12-OH BA ratio was lower in both the MT and HFD groups (*p* < 0.01 and *p* < 0.001, respectively) compared with the NCD control group (Fig. 4D).

### mRNA expression levels of liver and ileal BA receptors are changed after MT

**Fig. 5:**
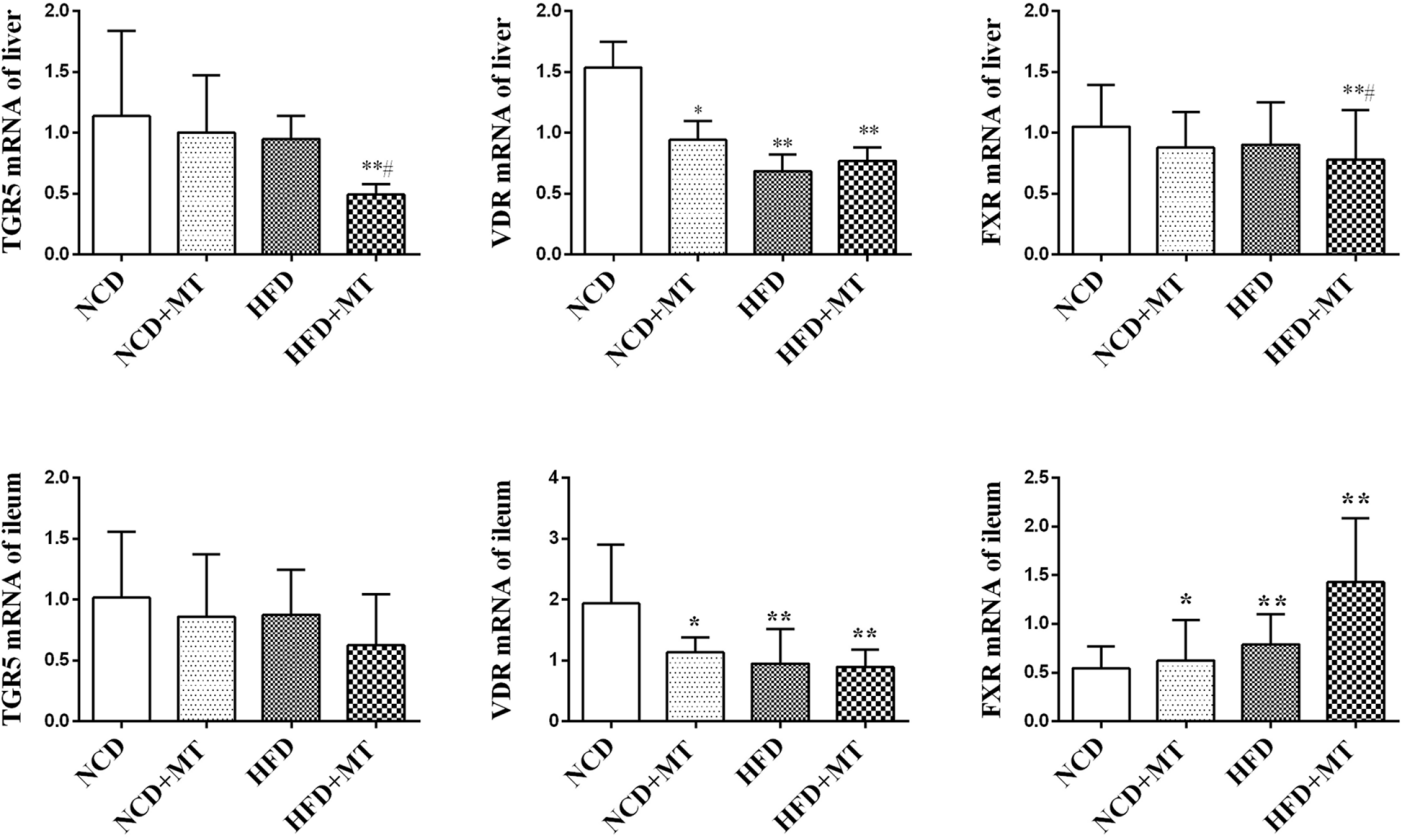
Effects of MT on liver and ileal BA receptors of mice. Results are expressed as the mean ± SEM (*n* = 7–8 per group). Compared with NCD group, ^*^P < 0.05, ^**^P < 0.01, ^***^P < 0.001. Compared with HFD group, ^#^ *P* < 0.05

As shown in Fig. 5, In the liver, the mRNA expression levels of VDR were decreased after MT and the HFD compared with the control group (*p* < 0.05 and *p* < 0.01, respectively), but the TGR5 and FXR mRNA levels were not significantly altered. Compared with the HFD group, the expression levels of TGR5 and FXR mRNA were significantly lower in the HFD + MT group (both *p* < 0.05). In the ileum, the mRNA expression levels of VDR were also decreased after MT and the HFD compared with the control group (*p* < 0.05 and *p* < 0.01, respectively). The mRNA expression levels of FXR were increased after MT and the HFD compared with the control group (*p* < 0.05 and *p* < 0.01, respectively).

### Protein expression levels of liver and ileal VDR decrease after MT

**Fig. 6:**
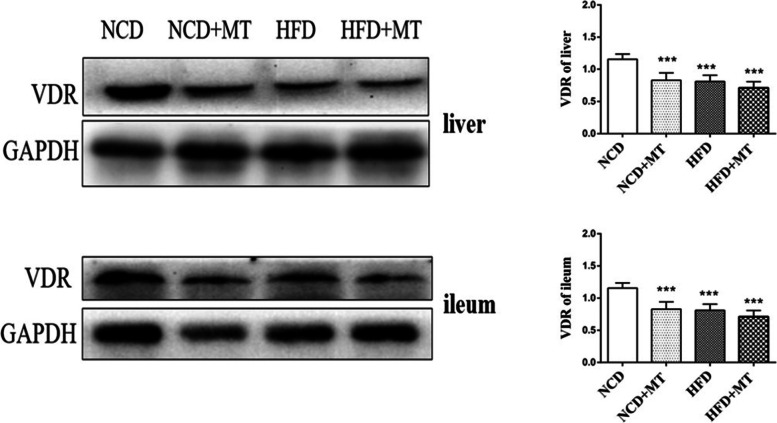
Effects of MT on liver and ileal VDR protein in mice. Results are expressed as the mean ± SEM (*n* = 4 per group). Compared with NCD group, ^*^P <0.05, ^**^P <0.01, ^***^P <0.001

As shown in Fig. 6, MT and the HFD significantly decreased the protein expression of VDR in the ileum and liver (*p* < 0.01 and *p* < 0.001, respectively) compared with the control group.

### Ileal GLP-1 decreases after MT

**Fig. 7:**
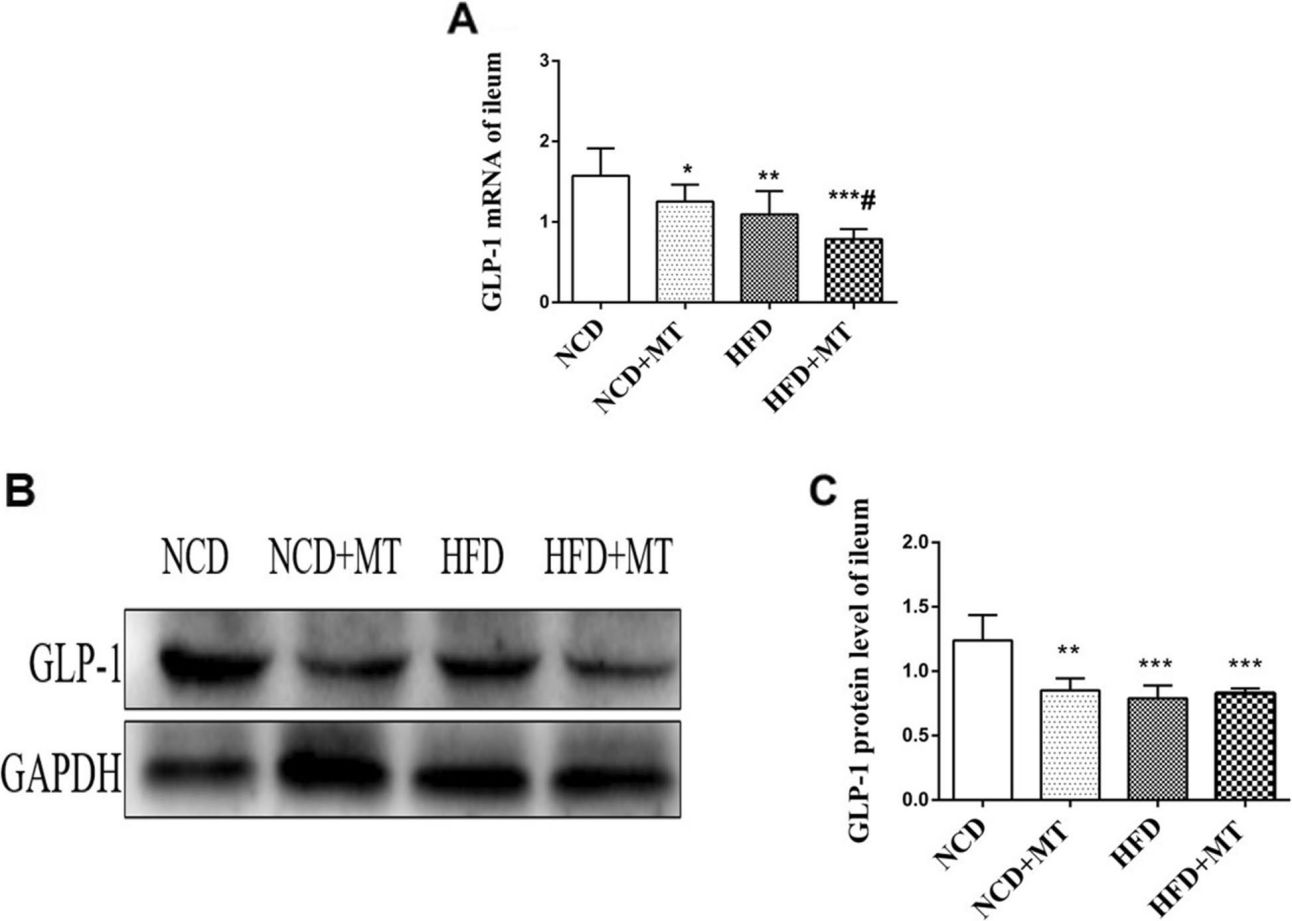
Effects of MT on ileal GLP-1 protein in mice. Results are expressed as the mean ± SEM (*n* = 4 per group). Compared with the NCD group, ^*^p < 0.05, ^**^p < 0.01, ^***^p < 0.001. Compared with the HFD group, ^#^*p* < 0.05

The mRNA expression levels of GLP-1 in the ileum were significantly lower (*p* < 0.05 and *p* < 0.01, respectively) in the MT and HFD groups compared with the control group. In addition, compared with the HFD, MT significantly decreased the mRNA expression level of GLP-1 in the ileum (*p* < 0.05). The protein test results showed that both MT and the HFD decreased the protein expression of GLP-1 in the ileum (*p* < 0.01 and *p* < 0.001, respectively) compared with the control group (Fig. 7).

## Discussion

Multiple factors influence the development of T2DM, including genetics, lifestyle and the gut microbiome [22–24]. Current evidence supports the influence of the microbiome on obesity and T2DM. For example, fecal microbial metagenomes of 145 European women with normal glucose control, impaired glucose and diabetes were characterized by shotgun sequencing, and a mathematical model based on the subgenomic map was established that could identify T2DM with high accuracy [25]. A study based on Pearson correlation coefficient analysis showed that bodyweight was significantly negatively correlated with the α diversity of the intestinal flora [26]. Another study found that the proportion of *Firmicutes* to *Bacteroidetes* was increased in the intestinal flora of obese patients [27]. In our study, principal component analysis and nonmetric multidimensional scaling revealed that MT and a dietary intervention altered the composition of the intestinal flora. MT and a HFD both significantly increased the *Firmicutes* to *Bacteroidetes* and *Clostridiales* to *Bacteroidales* ratios.

A study using a CA intervention in mice with a normal diet found that higher amounts of CA were transferred into DCA in the cecum of mice and that the changes in their intestinal flora were similar to those in mice fed a HFD. The CA intervention increased the proportion of *Firmicutes* from 54.1 to 93.4% and significantly increased the *Firmicutes* to *Bacteroidetes* ratio; the increase in *Firmicutes* was mainly caused by an increase in *Clostridia* and *Erysipelotrichi* [28]. Another study reported that a HFD significantly decreased *Bacteroidetes* and increased *Firmicutes* and *Proteobacteria* [29]. Our results showed that MT increased the *Firmicutes* to *Bacteroidetes* ratio and *Clostridia* and *Erysipelotrichi* in the intestinal flora. To test whether the ability of fecal bacteria to metabolize BAs had changed, we conducted in vitro co-culture experiments involving fecal bacteria and a BA substrate. The results showed that MT significantly increased the ability of fecal bacteria to metabolize the primary BA CA to produce DCA and decreased the ability of fecal bacteria to metabolize CDCA to produce LCA. An LC-MS assay was further used to detect the levels of circulating BAs in mice, finding that MT raised the DCA level and lowered the T-β-MCA level and the non-12-OH BA:12-OH BA proportion in plasma. These results suggested that BAs are closely related to the intestinal flora and are important metabolites of the intestinal flora.

Further work reported that downregulation of liver CYP8B1 increases the MCA:CA ratio (non-12-OH:12-OH) in BAs and improves lipid metabolism [30]. The Jia Wei research team [31] pointed out that BAs act as a signaling molecule to activate a variety of nuclear and membrane receptor pathways and play a role in regulating glycolipid, inflammation and energy consumption, thereby affecting metabolism-related diseases such as obesity, T2DM and non-alcoholic fatty liver disease, in which activation of the BA synthesis substitution pathway leads to an increased proportion of non-12-OH BAs. This would help to improve glucose and lipid metabolism. Other studies have found that the CYP8B1-derived 12-OH BA increase is associated with human insulin resistance and metabolic disorders such as T2DM [32].

Plasma and fecal BA levels of 297 obese patients recently indicated that plasma BAs showed considerable variability but were largely consistent with fecal BAs, confirming that BA levels are determined by potential genetic and microbial factors [33]. The 12α-OH:12α-non-OH BA ratio in plasma and feces was positively correlated with the presence of diabetes. The Jia Wei [34] team measured the levels of BAs and the intestinal flora in 121 healthy and 62 unhealthy individuals with a high BMI and found that the non-12-OH BA levels were significantly lower in the non-healthy participants with a high BMI. A decrease in the non-12-OH BAs in fat-prone mice fed a HFD was related to the secretion of GLP-1 in the ileum and germ-free mice showed obesity resistance induced by HFD and significantly lower levels of non-12-OH BAs than normal-fed mice [35]. Other studies have determined that antibiotic treatment increases the T-β-MCA level and reduces HFD-induced glucose intolerance [36]. Metformin is a first-line drug for T2DM and Sun et al. [37] found that metformin treatment decreased the abundance of *Bacteroides fragilis* and increased the level of GUDCA in the intestine, thereby inhibiting intestinal FXR signaling and the synthesis of liver glycogen. Recently, the Wang Weiqing team [38] conducted a randomized, double-blind, controlled trial on 409 patients with T2DM and speculated that berberine might exert hypoglycemic effects by inhibiting the production of the secondary BA DCA by *Ruminococcus bromii*. A study of antibiotic administration to HFD mice found that it reversed the HFD-induced high CA and DCA status and improved insulin sensitivity [16]. Another study showed that antibiotics antagonized FXR by elevating T-β-MCA, resulting in a reduction in insulin resistance [18]. Bile diversion to the ileum (GB-IL) induced weight loss and improved oral glucose tolerance in TGR5^−/−^, but not FXR^−/−^, mice fed HFD. The results also showed that the glucoregulatory effects of GB-IL were lost in whole-body GLP-1r^−/−^ mice [39], which indicated that BAs improve glucose homeostasis via an intestinal FXR–GLP-1 axis.

## Conclusions

Uygur T2DM fecal microbiota transplantation disrupts glucose metabolism and its possible mechanisms is to change the structure of the intestinal flora and then change the ability of intestinal flora to metabolize BAs and affect the BAs/GLP-1 pathway.

## Supplementary Information

**Additional file 1.**

## Data Availability

The datasets generated and/or analyzed during the current study are not publicly available due to institutional policies and regulations but are available from the corresponding author on reasonable request.

## Abbreviations

BAs: bile acids
CA: cholic acid
DCA: deoxycholic acid
FXR: farnesoid X receptor
GCA: glycocholic acid
GCDCA: glycogodeoxycholic acid
GLP-1: Glucagon-like peptide-1
HFD: high-fat diet
LCA: lithocholic acid
MT: microbiota transplantation
NCD: normal chow diet
OTU: operational taxonomic unit
RT-qPCR: real-time quantitative PCR
T2DM: Type 2 diabetes mellitus
TCA: taurocholic acid
TCDCA: taurochenodeoxycholic acid
TGR5: G-protein coupled receptor 5
T-β-MCA: tauro-β-muricholic acid
UDCA: ursodeoxycholic acid
UPLC-MS: Ultra Performance Liquid chromatography- mass spectrometry
VDR: vitamin D receptor
