# Supplementary material for: Uygur type 2 diabetes patient fecal microbiota transplantation disrupts blood glucose and bile acid levels by changing the ability of the intestinal flora to metabolize bile acids in C57BL/6 mice
Source: BMC Endocr Disord. 2022 Sep 23;22:236. doi: 10.1186/s12902-022-01155-8 (PMC9503279; doi:10.1186/s12902-022-01155-8)
Supplement: Supplementary file 1 — Additional file 1. [file 12902_2022_1155_MOESM1_ESM.pdf]

Uygurs Type 2 diabetes patient fecal microbiota transplantation disrupts blood glucose and bile acid levels by changing the ability of the intestinal flora to metabolize bile acids in C57BL/6 mice

Chanyue Wang[a], Ye Wang[a], Hao Yang[a], Zirun Tian[a], Manli Zhu[a], Xiaoting Sha[a], Ju Ran[a], Linlin Li[a,b,c*]

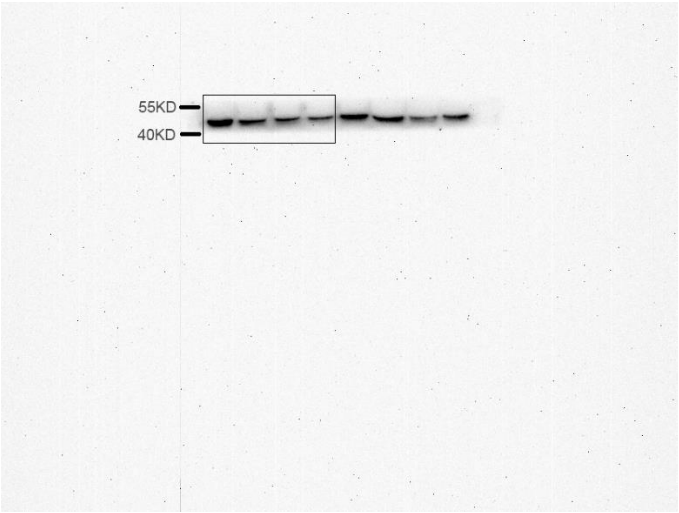

original full-length blot of Figure 8 liver VDR

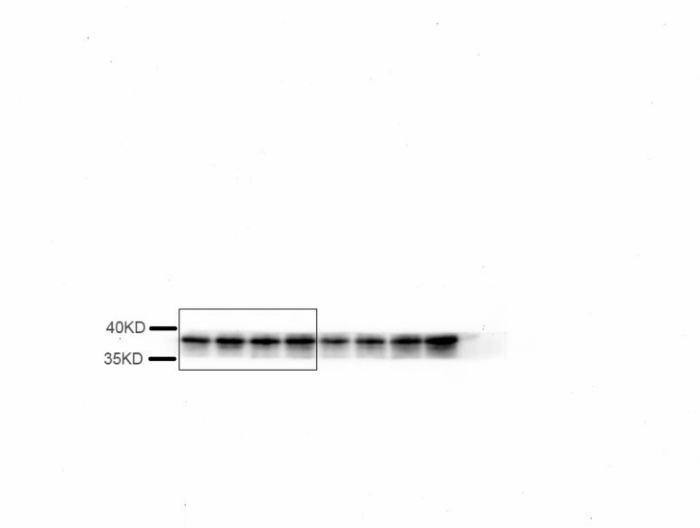

original full-length blot of Figure 8 liver GAPDH

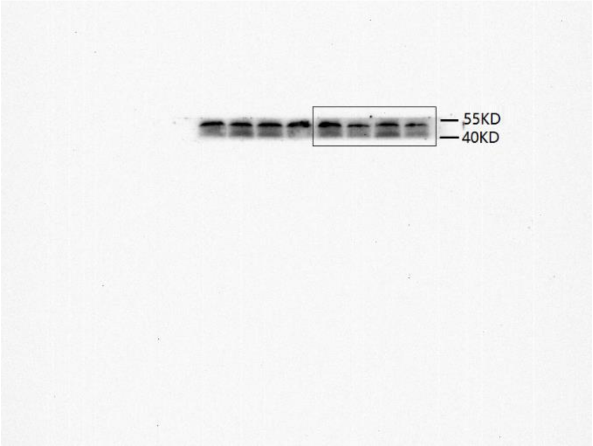

original full-length blot of Figure 8 ileum VDR

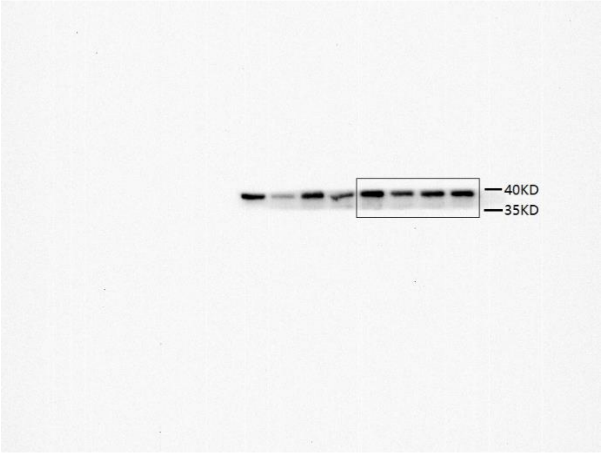

original full-length blot of Figure 8 ileum GAPDH

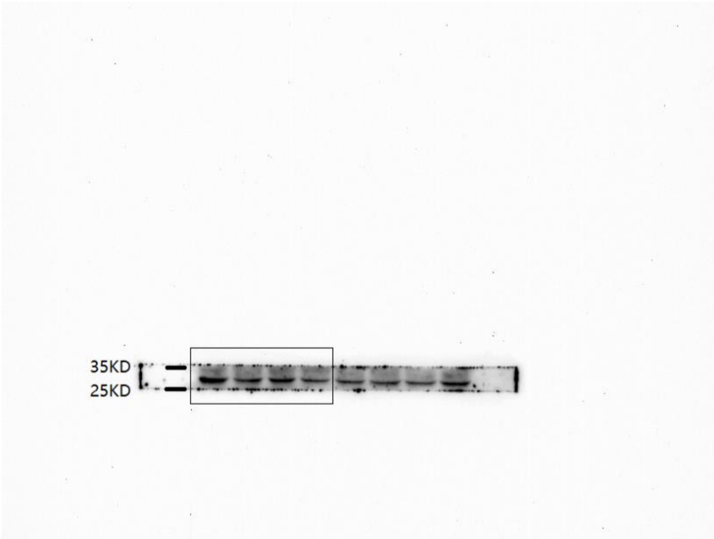

original full-length blot of Figure 9 ileum GLP-1

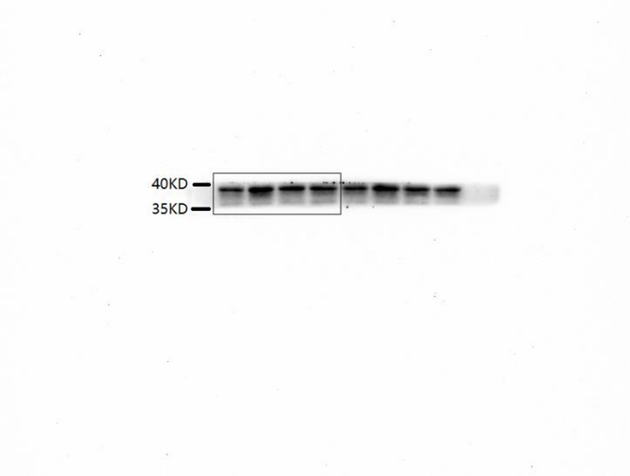

original full-length blot of Figure 9 ileum GAPDH
